# Chronic constipation in the elderly: a primer for the gastroenterologist

**DOI:** 10.1186/s12876-015-0366-3

**Published:** 2015-10-14

**Authors:** Roberto De Giorgio, Eugenio Ruggeri, Vincenzo Stanghellini, Leonardo H. Eusebi, Franco Bazzoli, Giuseppe Chiarioni

**Affiliations:** 1 Department of Medical and Surgical Sciences/Digestive system, University of Bologna and St. Orsola-Malpighi Hospital, Bologna, Italy; 2 Division of Gastroenterology of the University of Verona, AOUI Verona, Verona, Italy; 3 UNC Center for Functional GI & Motility Disorder, University of North Carolina at Chapel Hill, Chapel Hill, NC USA; 4 Division of Gastroenterology of the University of Verona, Ospedale Policlinico GB Rossi, Piazzale LA Scuro, 10, 37134 Verona, Italy

**Keywords:** Elderly, Fecal impaction, Irritable bowel syndrome, Constipation, Laxatives

## Abstract

Constipation is a frequently reported bowel symptom in the elderly with considerable impact on quality of life and health expenses. Disease-related morbidity and even mortality have been reported in the affected frail elderly. Although constipation is not a physiologic consequence of normal aging, decreased mobility, medications, underlying diseases, and rectal sensory-motor dysfunction may all contribute to its increased prevalence in older adults. In the elderly there is usually more than one etiologic mechanism, requiring a multifactorial treatment approach. The majority of patients would respond to diet and lifestyle modifications reinforced by bowel training measures. In those not responding to conservative treatment, the approach needs to be tailored addressing all comorbid conditions. In the adult population, the management of constipation continues to evolve as well as the understanding of its complex etiology. However, the constipated elderly have been left behind while gastroenterology consultations for this common conditions are at a rise for the worldwide age increment. Aim of this review is to provide an update on epidemiology, quality of life burden, etiology, diagnosis, current approaches and limitations in the management of constipation in the older ones to ease the gastroenterologists’ clinic workload.

## Background

Chronic constipation is the prototype of functional gastrointestinal disorders (FGID) and a condition frequently encountered in clinical practice, both in specialty office (e.g., gastroenterology, geriatrics) and in general medicine.

About 30 % of the general population experiences problems with constipation during life time [1, 2], with elderly people and women being mostly affected. However, only a minority of patients (approximately 25 %) uses medical treatments, whereas a considerable proportion relies on alternative solutions, following advices given in pharmacies or herbalist’s shops [1].

As for other FGID, such as functional dyspepsia or irritable bowel syndrome (IBS), chronic constipation has considerable impact on health expenses and quality of life [3, 4]. Moreover, constipation can be associated with relevant comorbidities. In the elderly, constipation is significantly associated with lower urinary tract symptoms commonly improved by the restoration of regular bowel movements [5]. In addition, constipation may lead to faecal impaction and, although rarely, proceed to stercoraceous perforation of the colon, a life threatening disease [6, 7]. Significant comorbidities are of particular relevance in hospitalized or bedridden elderly patients, who often have associated neurodegenerative diseases such as Alzheimer’s or Parkinson’s disease [7].

One of the conceptual aspects that makes constipation a clinical entity arduous to be managed is the absence of a generally accepted definition of the disease. The traditional criterion refers to a limited number of weekly evacuations, although patients mostly complain of symptoms associated with difficult stool passage, such as evacuation of hard or lumpy stools or the need of excessive efforts and/or manipulation during defecation [2].

Epidemiological studies confirm that about 2–3 % of the general population report a lower than normal number of evacuations (<3 times) per week [8]. However, this criterion tends to underestimate the considerably large amount of patients actually suffering from this condition [2].

Aim of this review is to define epidemiological, clinical and pathophysiological features of chronic constipation, as well as to evaluate the impact of this condition on quality of life with a particular focus on elderly subjects. An update on the management of constipation in the elderly is also provided.

## Review

### Epidemiology

Similarly to most FGID, chronic constipation is more commonly diagnosed in female patients (M/F ratio: 1: 2–3) (1, 2). Constipation is a prevalent disorders in Western countries that may affect up to 30 % of the general population subject to the caveats that definitions of constipation vary across studies (2). The financial burden of chronic constipation is considerable, due to direct costs of the healthcare system such as consultations, investigations, and drug therapy [9].

About 85 % of constipated patients that require medical care are already using laxatives and, every year, in the United States approximately 82 million dollars are spent for over-the-counter laxatives [10, 11].

Variability across studies on the prevalence of constipation is due to several factors, including the age of the population under investigation, the definition of constipation used and “those who propose it” (i.e., reported by patient or by a health care professional), as well as the context in which the studies are carried out (i.e., community people or hospitalized patient). The prevalence of constipation increases with age: in over 65 year-old population studies, 26 % of women compared to 16 % of men considered themselves to be constipated, while in a 84 year-old subgroup of patients, the proportion of sufferers increased to 34 % in women and 26 % in men, thus showing that age apparently leads to a substantial levelling between sexes [12, 13]. Moreover, when the prevalence of self-reported constipation was investigated in a door-to-door survey of 209 community-dwelling elderly 30 % of men and 29 % of women described themselves as constipated at least once a month [14]. The primary symptom used to define constipation was having to strain in order to defecate. The number of chronic illnesses and the number of medications were significantly related to constipation [14]. In addition, frailty in older persons is very common and is associated with immobility, poor food intake, and dehydration [15]: an old study reported that constipation is present in 45 % of frail elderly persons [16]. In a community-based study from Olmsted County (Minnesota, USA), which included 100 patients aged 65 years-old or older, the overall prevalence of constipation reported by patients was 40 %: 24.4 % affected by functional constipation and 20.5 % by outlet dysfunction [17]. In a recent study, data from the Australian Longitudinal Study of Ageing were used to compare differences in constipation and laxative use in 239 community elderly between 1992–93 and 2003–04. Over the years, the prevalence of self-reported constipation increased from 14 to 21 % as well as laxative use from 6 to 15 %. Persistent chronic constipation was reported by 9 % of the cohort. Female prevalence was evident at both time points. Unexpectedly, the association between laxative use and self-reported constipation was poor (less than a third of cases) suggesting sub-optimal management of constipation in the elderly [18].

When defining constipation on the basis of the number of weekly bowel movements, its prevalence decreases to values of 10 % or lower using a cut-off of more than 2 evacuations or less per week. Interestingly, among people who report constipation, only up to 10 % have less than two bowel movements per week while almost half of them have a daily bowel movement [14, 19, 20].

Constipation is more frequent among elderly patients forced to periods of long-term care in hospital or nursing homes [21, 22]. A Finnish study showed a prevalence of constipation or evacuation disturbances in 57 % of women and 64 % of men among the general population whereas the prevalence increased to 79 and 81 %, respectively, among guests of a nursing home [23]. Moreover, up to 74 % of patients staying in long-term care facilities uses laxatives on a daily basis [23]. A recent prospective study on elderly inpatients aimed to explore predictors associated with constipation during acute hospitalization comparing stroke patients (*n* = 55) with orthopaedic patients (*n* = 55) [23]. The incidence of “de novo” constipation was high for both stroke (33 %) and orthopaedic patients (27 %; *p* = 0.66) with bedpan use and longer length of stay both increasing new-onset constipation [24]. The high rate of constipation in the elderly population not only results in worsening of quality of life and incremental economic costs, but can also increase the risk of several complications including overflow faecal incontinence thus prolonging hospitalization [20–22].

### Quality of life

It is generally assumed that constipation affects unfavourably patients’ quality of life (QOL) [2, 3]. Rather unexpectedly, few solid data have been reported particularly in the elderly population. A recent study by Rao and co-workers analysed the effects of constipation on QOL and psychological status in 158 subjects with 76 having a functional defecation disorder and 38 slow colon transit constipation, while 44 were controls [25]. Subjects had to answer an 8-domain-questionnaire on health status, including general health, vitality, social functioning, emotional role (limitation of daily activities causing emotional problems) and mental health. A higher score was associated with a normal healthy status. Compared with patients with slow transit constipation and control subjects, patients with dyssynergic defecation had greater psychological distress and impaired health-related quality of life (HR-QOL) [25]. The latter group also showed a higher prevalence of paranoid ideation, hostility and obsessive-compulsive disorder compared to controls. Moreover, anxiety disorders, depression as well as somatization and psychosis had a significantly higher prevalence in both groups of patients with constipation symptoms compared to controls [25].

Similarly, in 126 community-dwelling older adults, respondents with chronic constipation had lower Short-Form 36 (SF-36) scores for physical functioning, mental health, general health perception, and bodily pain when compared to respondents without constipation [26]. Data were replicated using the Psychological General Well-Being (PGWB) index in 84 elderly subjects with constipation showing lower PGWB total scores and lower domain scores for anxiety, depression, well-being, self-control and general health subscales, indicating worse HR-QOL [3]. In addition, improvements on HR-QOL were noted with effective treatment of constipation. Increasing weekly bowel movements was associated with patients’ report of fewer urinary symptoms, better sexual function and improved mood [5]. More recently, in a study by Talley and co-workers, a questionnaire was proposed to 100 people over the age of 65 years in order to evaluate the impact of chronic constipation on their quality of life [27]. A markedly higher prevalence of physical pain and a decrease in perception of health in constipated patients compared to healthy controls was shown. The study also confirmed that constipation negatively affected both social and working life of patients [27].

Constipation is often associated with other symptoms that influence negatively the daily life. Indeed, an epidemiological survey of constipation performed in Canada showed that 32 % of constipated patients also need to make efforts during defecation, 20 % eliminate hard stools and 13 % has the feeling of incomplete evacuation or difficult stool passage [28].

### Pathogenesis of constipation

From a pathogenetic point of view, chronic constipation may itself be the disease, such as in primary forms, or be part of a complex clinical picture, as in secondary forms. This distinction is crucial for a proper management of constipation.

Primary forms are further distinguished according to their pathophysiological characteristics:

1) *Slow transit constipation* is characterized by prolonged transit time of stool through the colon and an often reduced rectal sensitivity. In physiological conditions colon motor activity is irregular, since it increases after meals and after wake up while it decreases during sleep [29]. It is characterized mainly by not propagated waves, that allow the mixing of intraluminal content in order to promote water and electrolytes absorption, and also by propulsive waves, including high (HAPCs) and low amplitude propagated contractions (LAPCs) [29–31]. HAPCs promote rapid movements of intraluminal content and their presence is often associated with evacuation [29].

Patients suffering from chronic constipation showed a significantly HAPCs reduction (<5 per day) compared to healthy controls [29–31]. In addition, gastro-colic reflex, which exerts an important control on colonic peristalsis, is deficient in patients with chronic constipation [32]. Thus, altered colonic motility plays a major role in the slowdown of gastrointestinal transit in patients with slow-transit constipation.

Slow transit constipation may be associated with several endocrine and metabolic disorders, such as hypothyroidism, hypercalcemia, porphyria or diabetes mellitus, or may occur without any other significant systemic, gastrointestinal or neurological diseases [2].

Recent studies on slow transit constipation aimed to define alterations involved in cellular mechanisms of intestinal coordination and motor function, such as smooth muscle innervation (intrinsic or extrinsic) and interstitial cells of Cajal (ICC, the pace-maker of gastro-entero-colonic motility) [33]. In particular, histological studies of biopsy specimens obtained from patients who underwent colectomy for severe constipation have shown alterations in both enteric neurons (apoptotic type which justify a greater tendency to neurodegeneration) and enteric glial cells (cells that support enteric neurons), leading to neuronal survival impairment; indeed, glial cells produce neurotrophic factors, the lack of which could act as a trigger signal of neurodegeneration [33–35]. Enteric neuronal function abnormalities are associated with a reduced amplitude of nerve inhibitor impulses on colon circular muscular layer and, hence, with a lack of coordination between colonic segments [32].

Furthermore, surgical specimens obtained from patients undergoing colectomy for severe constipation have shown a marked depletion of ICC [36]; how and why these pacemaker cells alterations can influence neuro-mediated mechanisms remains unclear in the pathophysiology of slow transit constipation.

Colonic structural abnormalities can include neuropathies/myopathies/*mesenchymopathies*, if ICC are affected, or can be often combined (neuro-ICC-myopathies); furthermore, the resulting dysfunction may diffusely involve alimentary canal [36]. In this case constipation is part of a generalized gastrointestinal motility disorder such as that of chronic intestinal pseudo-obstruction [33, 36].

Two relevant aspects of slow transit constipation pathophysiology have been noticed specifically in elderly: an increased deposit of collagen in the ascending colon, which may cause both motor and compliance alterations [34], and the presence of a greater number of binding sites for plasmatic endorphins [37]. Both these mechanisms, although apparently not connected to each other, may contribute to slowing down the faecal transit, leading to constipation.

2) *Outlet obstruction*: (*constipation by difficult or unsatisfactory expulsion of faeces from rectum)* may result from a lack of coordination between abdominal muscles contraction and pelvic floor muscle relaxation on straining, and/or from an obstructed perineal transit due to anorectal structural abnormalities or uro-gynecological diseases [2].

The anal sphincter pressure reduction, both at rest and on squeezing, may be caused by loss of muscular mass and contractility along with a damage of the pudendal nerve [17]. In particular, in the elderly it is also associated with a lower elasticity of the rectal wall, with a fibro-adipose degeneration and with an increased thickness of the internal anal sphincter [38, 39]. Therefore, during events such as anal stenosis or fissures, proctitis, rectocele, haemorrhoids and uro-gynecological disorders pelvic floor dysfunction may develop causing both faecal incontinence and constipation on the aged anorectum [39].

3) *Constipation in IBS*: in this case the typical symptom is abdominal pain that tends to resolve or markedly fade with evacuation. Although IBS is more common in younger individuals, elderly subjects are not spared by this FGID and the diagnosis might be overlooked [40]. A recent survey of 230 consecutive elderly attending an elderly care clinic, showed that symptoms suggestive of IBS were reported by 22 % of the sample often associated with disabling non-colonic symptomatology [40]. However, a physician diagnosis was only made in one patient without taking the chance of reducing the overall burden of suffering in those potentially affected [40].

Different types of primary constipation may be present alone or coexist in the same patient [41].

On the other hand, secondary forms of constipation can be caused by several systemic diseases as well as by some drugs of common use, including opiates, anticholinergics, calcium channel blockers and NSAIDs [2]. The most common causes of secondary constipation are summarized in (Table 1).

**Table 1:** Common causes of secondary constipationᅟ

Drugs	Anabolic steroids, analgesics, opioids (codeine), NSAIDs, anticholinergics, anticonvulsivants, antidepressants, antihistamines, antihypertensives (verapamil e clonidine), anti-Parkinsonian, diuretics, antiacids containing calcium or alluminium, cholestyramine.
Neuropathic and myopathic disorders	Amyloidosis, Chagas disease, connective tissue disorders, CNS lesions, autonomic diabetic neuropathy, Hirschprung’s disease, multiple sclerosis.
Idiopathic	Paraneoplastic syndromes, Parkinson’s disease, dementia, scleroderma, post-viral colon-paresis, intestinal pseudo-obstruction, spinal or ganglion tumor, ischemia.
Electrolytic balance alterations	Hypokalemia, hypercalcemia
Organic intestinal diseases	Obstruction/stenosis: adenoma, cancer, diverticolitis, rectocele, hernia, foreign bodies, faecal impaction, IBD and complications.
	Anorectal abnormalities: anal stenosis or fissures, proctitis, rectocele, haemorrhoids.
	Hypothyroidism, diabetes mellitus, pregnancy and childbirth, dehydration, low fibres intake diet, hyperglycemia
Endocrine-metabolic causes	

Constipation may be induced by all conditions that alter the integrity of both structural and functional components of neuro-muscular bowel system such as amyloidosis, Hirschsprung’s disease and diabetes mellitus neuropathy, neurodegenerative diseases (Alzheimer’s disease, PD and tauopathies in general) and paraneoplastic syndromes [2, 12]. Thus, since the prevalence all these conditions increases with age, also does constipation [12, 22]. Moreover, dyssynergic defecation with outlet dysfunction is relevant aetiology for constipation in PD and correlates with the severity of the neurology disease [42].

In addition, chronic constipation may occur in patients suffering from psychological/psychiatric disorders, endocrine abnormalities (especially hypothyroidism), and hydroelectrolytic abnormalities, such as hypokalemia and hypercalcemia, all common conditions in the elderly [22].

Finally, elderly patients often live a sedentary lifestyle, reduce water intake resulting in dehydration, and eat less fibre in their diet affecting gastrointestinal transit and promoting constipation [14, 22]. We have recently reported on the incidence of new onset constipation in six out of ten healthy young males with symptoms suggestive for outlet dysfunction after 1 month of experimental bed rest [43].

### Diagnosis

In clinical practice, dealing with a constipated patient requires accurate collection of anamnestic data, with particular attention to family history, medications (especially those that are known to slow down the gastrointestinal transit) and comorbidities, together with a physical examination that includes digital rectal examination [2].

In an effort to improve diagnostic categorization, an International group of experts has proposed a number of symptom-based criteria for FGID, including chronic constipation, known as the Rome criteria. In Table 2 are listed the main criteria as reported in the latest edition (Rome III) [41]. These, together with the exclusion of alarm symptoms (such as rapid weight loss, hematochezia, family history of colorectal cancer or inflammatory bowel disease, positive faecal occult blood test, iron deficiency anaemia and a recent onset constipation) may lead to the diagnosis of functional constipation, often avoiding unnecessary and costly examinations. The presence of any alarm symptom requires further investigations, including colonoscopy.

**Table 2:** Rome III diagnostic criteria for chronic constipation

1-MUST INCLUDE TWO OR MORE OF THE FOLLOWING:
a. Straining during at least 25 % of defecations
b. Lumpy or hard stools in at least 25 % of defecations
c. Sensation of incomplete evacuation for at least 25 % of defecations
d. Sensation of anorectal obstruction/blockage for at least 25 % of defecations
e. Manual maneuvers to facilitate at least 25 % of defecations (e.g., digital evacuation, support of the pelvic floor)
f. Fewer than three defecations per week
2-LOOSE STOOLS ARE RARELY PRESENT WITHOUT THE USE OF LAXATIVES
3-INSUFFICIENT CRITERIA FOR IRRITABLE BOWEL SYNDROME

While the diagnostic predictivity of Rome III criteria in irritable bowel syndrome-related constipation has been evaluated in several studies, data on chronic constipation are still lacking [44]. In addition, although often applied in clinical trials the Rome III criteria are not commonly used in the clinical practice [44]. O the other hand, the Bristol stool scale could be a useful tool in daily practice [45]. This is a seven level scale based on the texture degree and morphology of faeces, which correlates with gastrointestinal transit times. The first two levels are representative of slow intestinal transit, while stool consistency levels of 6 and 7 correlate to an accelerated transit and diarrhea [45].

A well performed digital rectal examination may reveal the presence of morphology alterations of the pelvic floor (proctitis, rectal prolapse, rectal cancer, etc.), additionally allowing for functional evaluation of the anorectum (anal sphincter tone, evacuatory dysfunction) [2, 7].

Furthermore, rectal examination is highly relevant on diagnosing faecal impaction, a major cause of bowel obstruction in the elderly [7]. In some cases, faecal impaction can induce pseudo-diarrhoea due to the passage of fluid and mucus around the faecal conglomerate (*overflow*) [7]. If clinically misinterpreted, pseudo-diarrhoea can lead to the administration of anti-diarrheal drugs that further aggravate intestinal obstruction [7]. Rectal examination can be integrated with the use of an anoscope or a proctoscope, which allows a direct view of anal canal and rectum [2]. A simple Foley catheter balloon expulsion test has been proved reliable and useful test to diagnose an evacuation disorder of both functional and altered morphology origin [46]. In addition, to obtain a complete evaluation a standard abdominal radiograph and a barium enema could eventually be considered to look for megabowel and/or massive stool retention [47].

In the absence of alarm signs, a correct management of constipated patients is based on the use of an empiric therapy, followed by the observation of clinical effects, which can lead the clinician to a specific diagnosis [47]. In non-responders to conservative treatment, some functional tests are useful to clarify pathophysiological changes in patients with constipation after consideration of performing endoscopy.

A gastrointestinal transit time evaluation, which consists in ingesting radio-opaque markers, followed by an abdominal radiological evaluation to check the distribution of markers, allows us to differentiate *slow transit constipation* (markers distributed along colic frame) from *outlet obstruction* (markers located almost exclusively in rectal ampulla) [31]. So far colonic manometry had a minimal clinical value and has been used only for research purposes [47]. The improvement of this test with the spatio-temporal map evolution, through high-resolution manometry, is expected to open new clinical perspectives to the manometric assessment of the colon [31].

Gastrointestinal and/or ano-rectal manometric tests may be clinically useful to reveal an underlying neuropathy or myopathy of gut, as well as to determine if motor patterns (inter-digestives and post-prandial) anomalies can be identified in the small intestine [33]. Some years ago, a study by our group clearly showed that approximately two thirds of patients with constipation had small intestine motor abnormalities [48]. Thus, proposing a colectomy to patients with severe slow transit constipation, without having evaluated the motility of both ano-rectum and small bowel by manometry, should be avoided. Indeed, the more the motor disorder is extended throughout the alimentary tract, the less is the long-term therapeutic success of colectomy in constipated patients [49]. In addition to standard techniques, colonic transit can also be measured using the wireless motility capsule (WMC) which simultaneously provides valuable transit time information about the stomach and small bowel as well. This becomes particularly relevant when a multi regional motility disorder is suspected and tolerance to invasive procedures is limited. A recent WMC study on 161 FGID patients has provided evidence of multiregional intestinal dismotility in approximately half of the subjects that was poorly gained by the clinical picture [50].

In *outlet obstruction*, anorectal manometry detects changes in the anal sphincters contraction and relaxation, related to the presence of faeces. These tests are important not only for diagnostic purposes but also to set therapies based on rehabilitation techniques such as bio-feedback [51].

Finally, dynamic videoproctography or MR defecography can be used to further investigate cases of constipation due to obstructed defecation [47]. Diagnostic strategies do not appear to depend upon age, but this needs to be evaluated on a case-to-case basis.

### Treatment

Non-pharmacological treatment, which consists in diet and lifestyle modifications is traditionally considered the first step of a comprehensive treatment program to effectively manage constipation. [47]. A number of patients would believe that they need to have a bowel movement every day; counselling on simple lifestyle changes may improve their perception of bowel regularity and a diary log reporting on stool pattern and consistency may be helpful as well [11]. In addition, patients should be educated on recognizing and responding to any urge to defecate. A regular daily routine, starting with a light physical activity, is particularly recommended. The optimal times to have a bowel movement are soon after waking and after meals, when normal colon accentuates its motor activity [29]. Therefore, patients should be advised to attempt defecation first thing in the morning and in the post-prandial intervals to profit most of the gastro-colic reflex [29].

A gradually increasing intake of fluids and fibres up to 30 g/day is suggested [11, 12]. This goal can be achieved by recommending patient to integrate the diet with more fruits, vegetables and nuts in addition to adding varying amounts of bran. However, in elderly patients, increments in fluid intake should be monitored especially in those with cardiac and renal disease [12]. In a classical study, this approach has been reported to fasten colon transit time in the constipated elderly without mirroring significant improvement on symptoms [52]. On the contrary, a recent small sized study reported on the efficacy of diet and lifestyle modification on symptoms and QOL in 23 constipated elderly showing a significant improvement on both parameters [53]. Moreover, the position paper of the American College of Gastroenterology on constipation had concluded that fibre are effective treatment in adults, but adverse events, bloating, distension, flatulence, and cramping may limit their use, especially if increases in fibre intake are not introduced gradually [54]. In addition, fibres appear to be scarcely useful not only in patients with proven slow transit constipation, but also in those who suffer from pelvic floor dysfunction [31]. Although very rarely, bowel sub-obstruction secondary to high fibres dietary intake have been reported in elderly patients [55]. In an effort to overcome bran side effects a number of soluble fibres have been developed among them: naturally occurring (psyllium seeds), semi-synthetic (methyl cellulose) and synthetic (calcium polycarbophil) [56]. These compounds might also be regarded as bulking forming laxatives for the mechanism of increasing stool bulk by holding liquid in the gut [56]. Psyllium and calcium polycarbophil have both been shown more effective than placebo in randomized controlled trials [56]. However, low palatability and occurrence of side effects, such as flatulence and abdominal bloating likely due to fermentation by intestinal microbiota have been associated with high drop-out rates in the elderly [57]. Finally, a recent randomized controlled trial (RCT) showed that dried plums were more effective than psyllium on improving bowel frequency and stool consistency in adults with mild to moderate constipation [58].

Other currently available non-pharmacological treatment options for constipation are probiotics. Nowadays, probiotics are familiar to the public as the components of bioyoghurts and dietary supplements, are widely available, and commonly prescribed. The faecal flora changes markedly with age mostly by a fall in numbers of bifidobacteria [55]. However, it is still unclear whether this is a cause or the effect of constipation. It has been repeatedly reported that probiotics in the elderly may both shorten bowel transit and soften stools most likely by the increased short chain fatty acid concentration [59]. A logical choice would be to consider probiotics as a mainstay of treatment for their lack of side effects and absence of inference with medications. Preliminary data supported this consideration, but large, randomized, controlled trials have failed to show a significant benefit on the complex clinical picture of constipation in the elderly [55, 59].

Biofeedback therapy to teach adequate defecatory effort is effective treatment in adults with dyssynergic defecation [51]. The treatment protocol employed in most RCTs performed in the adult population includes four steps: 1)Patient education on appropriate defecation effort, 2) Straining training to improve abdominal pushing effort, 3) Training to relax pelvic floor muscles while straining by visual feedback of anal canal pressure or averaged anal EMG activity, 4) Practice simulated defecation by using inflated rectal balloon [60]. Some Centers include optional sensory training which is intended to lower the threshold for the sensation of urgency to defecate [60]. In the older ones, data are limited to a single RCT reporting on clinical and anorectal physiology benefit associated with EMG-biofeedback treatment in 15 elderly patients with dyssynergia when compared to an analogue control group [61]. In community dwelling constipated elderly biofeedback might be considered a therapeutic option for dyssynergic defecation, but larger RCT are eagerly awaited [62].

Although widely practiced, stool softeners have limited evidence in the management of constipation in the elderly [62]. Suppositories and enemas may be used in institutionalized patients to help rectal evacuation in an effort to prevent faecal impaction [22]. Side effects such as electrolyte imbalances and rectal mucosal damage have been reported with the use of phosphate and soapsuds enema, respectively. When indicated, tap water enema is the safest way to go [22].

### Pharmacological therapy

Usually, when simple changes to lifestyle and diet do not improve constipation, the use of laxatives is recommended [62]. However, the use of laxatives must be individualized with special attention to cardiac and renal co-morbid conditions, drug interactions, and side effects particularly in the frail elderly [22, 62]. This heterogeneous group of drugs includes many products which differ in pharmacological characteristics and mechanism of action, but all of them having the common purpose of stimulating defecation or softening the consistency of faeces in order to facilitate their expulsion (Table 3). Many types of laxatives are nowadays available, however we will focus mainly on those with major indications for the treatment of chronic constipation in the elderly.

**Table 3:** Laxative compounds commonly used to treat chronic constipationᅟ

Type	Laxative agent	Mechanism of action	Possible side effects
Bulking forming laxatives	Natural fibres (e.g., psyllium)	Intraluminal H_2_o binding, bulk forming and decrease stool consistency	Bloating, flatulence
	Semi-synthetic fibres (es. methylcellulose)		
	synthetic fibres (e.g., Polyethylene glycol polycarbophil: Macrogol)		
Osmotic laxatives	Magnesium hydroxide, magnesium citrate, magnesium sulfate, sodium phosphate.	Interstitial H_2_o binding	hydroelectrolytic alterations
Disaccharides and alditols	Lactulose, sorbitol.	Interstitial H_2_o binding	Bacterial fermentation with bloating and flatulence (low efficacy in *slow transit constipation*)
Emollients laxatives	Paraffin oil, docusate sodium	Intraluminal H_2_o binding, bulk forming and decrease stool consistency	*Discomfort*, abdominal pain, cramping
Stimulant laxatives	diphenylmethane derivatives (bisacodyl, sodium picosulfate)	Stimulating action on enteric nerves with decrease in peristaltic contractions.	*Discomfort*, abdominal pain, cramping
	Anthraquinones (senna, aloe, cascara)	Decrease in colic absorption of H_2_o and electrolytes	

Stimulant laxatives are a diverse class of agents derived primarily from anthraquinones and diphenylmethanes [31]. These drugs have a stimulating and irritating action on the intestinal mucosa that increases its secretory activity, thereby increasing water content in the intestinal lumen. In addition, these laxatives have probably a direct action on the enteric innervation (the enteric nervous system), increasing intestinal motor activity [55, 57]. To this class of laxatives belong senna, cascara, rhubarb, aloe, bisacodyl and sodium picosulfate [31]. Notwithstanding their limited cost, stimulant laxative chronic use has historically been discouraged based on anecdotal fears of potential complications [62]. In classical studies, silver staining studies suggested their chronic use may result in enteric neuropathies, including replacement of ganglia by Schwann cells and losses of neurons in the smooth muscle of the colon and myenteric plexus [31, 47]. More advanced techniques have failed to confirm these findings [31, 47]. Melanosis coli (dark colour of colonic mucosa) is a typical endoscopy finding in patients with prolonged use of stimulant laxatives, but it does not have a pathological significance [47].

In recent RCTs both bisacodyl and sodium picosulfate proved effective on increasing the number of complete spontaneous bowel movements/week compared to placebo in constipated adults, but data in the elderly are lacking [62]. However, a senna fibre combination in 77 constipated elderly residents in long term hospital or nursing home care improved stool frequency, stool consistency, and ease of evacuation when compared to lactulose [63].

Osmotic laxatives are hyperosmolar agents that cause secretion of water into the intestinal lumen by osmotic activity thus improving bowel transit and stool consistency. Lactulose, lactitol and macrogol are the most commonly and safest compounds used in the elderly [55, 57, 64].

Lactulose and lactitol are both synthetic, non-digestible disaccharides that are fermented by colonic bacteria to increase stool water content and soften the stool [57]. This process may enhance proliferation of lactobacilli (prebiotic action) and shorten bowel transit by promoting stool acidification [57].

In a multicenter trial of 164 patients including a group of elderly, lactulose was found to be more effective on improving bowel frequency by day seven compared with laxatives containing senna, anthraquinone derivatives, or bisacodyl [64]. In a similar study comparing osmotic compounds sorbitol was as effective as lactulose on improving constipation, but was cheaper and better tolerated [65]. Lactitol efficacy has been extensively evaluated in the constipated adults, but the efficacy in the elderly is limited [66]. A recent meta-analysis concluded that the efficacy on improving symptoms of constipation of lactitol and lactulose are similar as well as tolerance to the drugs [66]. Finally, a double blind vs placebo study conducted by Ouwehand et al. studied the effects of a symbiotic combination of Lactitol and *Lactobacillus acidophilus NCFM* on bowel function and immune parameters in a small group of healthy elderly [67]. The symbiotic preparation was more effective than placebo on increasing bowel movements and improving gut mucosal immune function, thus suggesting future therapeutic applications.

Among osmotic agents, polyethylene glycol (PEG) or macrogol 3350–4000 is the one where sound evidence of effectiveness on improving constipation in RCTs is best provided [54]. PEG is made from organic, iso-osmotic, non-absorbable polymers, that do not act by modifying osmotic exchanges but by retaining water introduced with diet within the intestinal lumen, hence increasing the faecal mass and reducing stool consistency [68]. Two pivotal RCTs, one in the US and the other in Europe, have shown that PEG is more effective than placebo on achieving long term treatment success in constipated adults [69, 70]. In the US based one, treatment success was defined as relief of modified Rome criteria for constipation for 50 % or more of weeks of treatment [69]. In this study the treatment effectiveness was similar when a subgroup analysis involving 75 elderly patients was performed [69]. In a large sized RCT, PEG 17 g daily was more effective than placebo at 4 weeks on improving drug induced constipation, a common problem in the elderly [71]. In a multicenter, placebo controlled study, PEG was shown to correct bowel movements also in IBS adult patients with constipation, but with no significant effects on digestive symptoms [72]. Bloating and flatulence represent the most frequent side effects of osmotic laxatives with some PEG studies failing to report on the total number of side effects [54]. A recent meta-analysis, showed that PEG, compared to placebo or to other laxatives (usually lactulose), significantly increased the number of bowel movements per week in constipated adults [73].

However, two recent reviews concluded that despite increasing efforts on including the elderly in RCTs, most studies on the use of laxatives in constipated older adults provide limited evidence for small sample size and methodology biases [57, 62]. In addition, severe laxative side effects as dehydration, electrolyte imbalances, allergic reaction, and hepatotoxicity have all been reported in the elderly suggesting a tailored approach in this potentially frail population [22].

Despite the wide range of laxatives, it is estimated that about half of constipated patients do not achieve satisfactory results with the drugs so far described [74]. Thus, new products based on more physiological mechanisms of action have been developed in the attempt to treat a larger share of constipated subjects [54, 62]. Among the new therapeutic options for constipation, emerging drugs in clinical practice include pro-secretory products (lubiprostone and linaclotide) and serotonergic agents (Table 4) [54, 74].

**Table 4:** New treatment options for laxative-resistant chronic constipationᅟ

Drug	Mechanism of action	Effect	Possible side effects
Lubiprostone	Type 2 chloride channel (CCl2) activator.	Chloride secretion in the intestinal lumen followed by passive diffusion of sodium and water.	Nausea, headache.
		Increase in faecal content of water with distension of intestinal walls and activation of peristalsis and acceleration of intestinal transit.	
Linaclotide Plecanatide	Guanylate cyclase C receptor agonist.	Increase of intra and extra-cellular cyclic guanosine monophosphate.	Dose-dependent diarrhea.
		Increase of secretion of chloride, bicarbonate and water into intestinal lumen.	
		Activation of peristalsis and acceleration of intestinal transit.	
Prucalopride	5-HT4 serotonin receptor agonist.	Excitatory activity of neurons of the myenteric plexus.	Headache, nausea, diarrhea.
Norcisapride		Release of acetylcholine.	
Velusetrag		Activation of peristalsis and acceleration of intestinal transit.	
Elobixibat	Enantiomer of 1,5-benzothiazepine.	Bond and inhibits the ileal bile acid transporter.	Abdominal pain, diarrhea.
		Increased stay of bile acid in the colon. Activation of peristalsis and acceleration of intestinal transit.	

Among drugs with intestinal secretagogue action, lubiprostone acts by activating type 2 chloride channel (CCl2), located in the apical membrane of enterocytes [75]. This effect determines chloride secretion in the intestinal lumen followed by passive diffusion of sodium and water [75]. It therefore causes an increase in faecal content of water, which increases the distension of intestinal walls with activation of the peristalsis, without having a direct effect on smooth muscle of digestive tract [76]. Lubiprostone at a dosage of 24 μg twice daily has been consistently shown in RCTs more effective than placebo on increasing number of spontaneous bowel movements (SBM) per week as well as improving stool consistency, straining, and constipation severity in the adult population [76–80]. The percentage of elderly patients included in the RCTs varied, but in one of these studies 10 % of the participants were elderly [78]. Moreover, data from three open-label clinical trials were combined to obtain a pool of elderly patients with chronic idiopathic constipation and published as abstracts suggesting similar benefit [80]. However, extrapolation of the results of clinical trials performed in the overall adult population to elderly patients must be done with caution and additional RCTs are warranted before confirming the efficacy of the treatment in elderly patients. While it does not cause electrolyte disturbances, lubiprostone evokes nausea (30 % of patients), and headache, probably due to its prostaglandin-like structure [77, 79]. Nevertheless, this drug appears to be tolerated more by older people since side effects appear to be less frequent than in younger people [80].

Linaclotide is a receptor agonist of guanylate cyclase C, located on the apical side of intestinal epithelial cells [81]. This drug causes an increase in intra and extra-cellular cyclic guanosine monophosphate, which is followed by an increase in secretion of chloride, bicarbonate and water into intestinal lumen, resulting in an activation of the peristalsis and acceleration of the intestinal transit [81]. The administration of linaclotide (150–300 micrograms a day) causes an increase in the number of complete SBM per week and reduces stool consistency and straining during defecation [81–84].

In a study by Rao and colleagues, among 1602 patients with severe abdominal symptoms (44 % of subjects had bloating, 44 % fullness, 32 % discomfort, 23 % pain and 22 % cramping, with considerable overlap among symptoms), 805 were treated with linaclotide while 797 received placebo [84]. In patients with severe symptoms, linaclotide reduced all abdominal symptoms; mean changes from baseline severity scores ranged from −2.7 to −3.4 for linaclotide vs −1.4 to −1.9 for placebo (*P* < .0001) [84]. Linaclotide improved global measures (*P* < .0001) and IBS-QOL scores (*P* < .01) compared to placebo [84]. In one of the pivotal study, 10 % of the whole sample where elderly subjects, who showed similar results in safety and improvement of weekly spontaneous bowel movements, stool consistency, straining, abdominal discomfort, and quality of life to the entire study population [83]. However, solid data on the efficacy and safety of Linaclotide in the constipated elderly are still lacking. The most common side effect is represented by a dose-dependent diarrhoea, but less than 5 % of patients are reported to discontinue treatment due to side effects [81–84].

After the withdrawal of cisapride from distribution in 2000 and the significant restrictions in the use of tegaserod (not marketed in Europe), new serotonergic drugs, including prucalopride, velusetrag and norcisapride, have emerged as effective new treatment options for chronic constipation [85]. To understand the mechanism of action of these drugs is necessary to analyse the basis of the physiological mechanisms of gastrointestinal motility [85]. Mechanical and chemical stimulation on the intestinal walls evokes peristalsis, a motor pattern fundamental for life of any living being with a gastrointestinal tract [85]. In fact, bolus (or endoluminal enteric content) evokes distortion/stimulation of enterochromaffin cells (cells containing a biogenic amine, serotonin or 5-hydroxytryptamine, 5-HT) that, distributed along the surface of digestive tract mucosa, react by secreting 5-HT. This mediator activates neural circuits that trigger peristalsis by binding to specific receptors at the level of enteric neurons (myenteric and submucosal plexus) [74, 85].

Among the seven types of serotonin receptors, 5-HT4 possesses a strong excitatory activity on neurons of the myenteric plexus causing release of acetylcholine and producing an increase in peristaltic movements [74, 85]. In this perspective, prucalopride is a high affinity agonist of 5-HT4 receptors, has high bioavailability and is not metabolized by cytochrome P3A4 which is associated with fewer interactions with other active ingredients, compared to other 5-HT4 receptor agonists [85]. The safety and efficacy of prucalopride in constipation has been evaluated in three large sized trials [86–88]. All studies were 12 weeks in duration with similar design: multicenter, randomized, double-blind, placebo-controlled, and parallel group [86–88]. To be included patients had to report infrequent defecation, hard stools and/or frequent straining resistant to laxatives. In all studies, the primary efficacy endpoint was the proportion of patients having three or more complete SBM per week, averaged over the 12-week period, using an intention-to-treat analysis. Secondary endpoints included average increase of one or more complete SBM per week, patient subjective satisfaction, QOL questionnaires, changes in bowel symptoms, stool consistency and straining at stool. All RCTs were concordant on reporting Prucalopride as effective treatment for chronic constipation in the adult population non responding to laxatives [86–88]. Most study participants were females, the preferred schedule was 2 mg/daily, since no differences in clinical efficacy were noticed between 2 and 4 mg schedules, the latter dose being associated with more frequent side effects including headache, nausea (usually mild and short-lived) and diarrhoea [86]. A *post-hoc* analysis showed that prucalopride not only favourably affects bowel movements, but also improves anorectal and abdominal symptoms including pain, bloating and distension [87]. The efficacy of prucalopride has also been tested short term in the constipated older ones [88]. Three hundred chronically constipated patients aged 65 years and over were randomized to receive prucalopride at the dosage of 1 mg, 2 mg, 4 mg or placebo once daily for four weeks. Approximately one third of study participants were males. Inclusion criteria, primary and secondary outcome parameters were the same as the pivotal studies run in the adult population [86]. Additional testing on cardiovascular function was performed. Prucalopride, in the dose range tested (1–4 mg once daily) was effective treatment for constipated elderly improving both symptoms and quality of life [88]. The lowest schedule was as effective as the 4 mg/day and the Authors speculated a potential drug clearing slower than in the adult population. The drug was safe and well tolerated with headache as the most commonly reported side effects (6.6 % on 1 mg schedule). In addition, the safety and effectiveness of Prucalopride have also been confirmed by a small sized RCT on 84 elderly nursing home residents with chronic constipation resistant to laxatives [89]. In both studies, prucalopride did not cause QT prolongation (reported in patients treated with cisapride) or other vascular disorders (i.e., ischemic colitis as rarely reported in tegaserod trials) [88, 89].

The new horizons in the treatment of chronic constipation include a variety of new compounds, such as other serotonergic drugs (velusetrag and norcisapride, both 5-HT4 receptor agonists) as well as molecules inhibiting the bile acid transporter (elobixibat) and a new guanylate cyclase-C agonist (plecanatide) [85, 90–94]. Velusetrag in a recent randomized-controlled trial study of 4 weeks was found to be effective and well tolerated in patients with chronic constipation [90], while in a pharmacodynamics study norcisapride has been shown to accelerate colonic transit in healthy volunteers [91]. Elobixibat, an enantiomer of 1,5-benzothiazepine, acts locally in the lumen of the gastrointestinal tract, binding and inhibiting the ileal bile acid transporter, thereby increasing bile acid content in the colon [85]. A randomized phase II placebo-controlled study with 3 different doses of elobixibat, demonstrated that the number of complete SBM raised progressively with the increase of the drug dosage compared to placebo [92]. Abdominal pain and diarrhoea were reported as main adverse events in this study [92]. Finally, similarly to linaclotide, plecanatide is a guanylate cyclase C agonist, which leads to secretion of fluids into the intestinal lumen, facilitating bowel movements [85]. A phase I study assesses the safety, tolerability, and pharmacokinetics of a single dose (ranging from 0.1 to 48 mg) of oral plecanatide in 79 healthy controls. Plecanatide was demonstrated to be safe and well tolerated, and no measurable systemic absorption of oral plecanatide was observed at any of the oral doses studied [93]. Moreover, a multicenter randomized trial compared 12 weeks of treatment with plecanatide (0.3, 1 or 3 mg daily) with placebo in 946 patients with chronic constipation. Plecanatide 3 mg was more effective than placebo on improving number of CSBM/week, stool consistency, straining and QOL score [94].

## Conclusions

In conclusion, constipation is a common, self-reported digestive symptom that affects up to 30 % of people in Western countries and has considerable impact on health expenses and quality of life. Older individuals are particularly prone to it with a reported prevalence of up to 50 % in community-dwelling elderly and up to 70 % in nursing-home residents. Loss of mobility, medications, associated comorbidities, and rectal sensory-motor dysfunction are as important as gut aging alterations in causing constipation. Digital rectal exam and clinical history may help on identifying causes of constipation, but more than one mechanism might be involved. Diet and lifestyle modifications are often ineffective to manage constipation in the elderly and a multifactorial approach is suggested. Laxatives remain a mainstay to solve the problem but safety concerns in the frail elderly should be addressed. In laxative resistant constipation, several new agents that target different underlying pathophysiological mechanisms have been proved to be safe and effective in adults, but only partially validated in the elderly. Additional RCTs addressing management of constipation in the elderly are needed to tailor treatment in this complex population and to improve the quality of life of these disabled patients.

## Abbreviations

FGID: Functional gastrointestinal disorders
IBS: Irritable bowel syndrome
QOL: Quality of Life
RCT: Randomized controlled trial
SBM: Spontaneous bowel movements
PEG: Polyethylene glycol

## References

[1] Higgins PD, Johanson JF (2004). Epidemiology of constipation in North America: a systematic review. Am J Gastroenterol.

[2] Bharucha AE, Pemberton JH, Locke GR (2013). American Gastroenterological Association technical review on constipation. Am Gastroenterol Assoc Gastroenterol.

[3] Glia A, Lindberg G (1997). Quality of life in patients with different types of functional constipation. Scand J Gastroenterol.

[4] Wald A, Scarpignato C, Kamm MA (2007). The burden of constipation on quality of life: results of a multinational survey. Aliment Pharmacol Ther..

[5] Charach G, Greenstein A, Rabinovich P, Groskopf I, Weintraub M (2001). Alleviating constipation in the elderly improves lower urinary tract symptoms. Gerontology.

[6] Berardi RS, Lee S (1983). Stercoraceous perforation of the colon. Report of a case. Dis Colon Rectum.

[7] Wrenn K (1989). Fecal impaction. N Engl J Med.

[8] Bharucha AE, Dorn SD, Lembo A, Pressman A (2013). American gastroenterological association medical position statement on constipation. Gastroenterology.

[9] Neri L, Basilisco G, Corazziari E (2014). Constipation severity is associated with productivity losses and healthcare utilization in patients with chronic constipation. United European Gastroenterol J..

[10] Dennison C, Prasad M, Lloyd A, Bhattacharyya SK, Dhawan R, Coyne K (2005). The health-related quality of life and economic burden of constipation. Pharmacoeconomics..

[11] Menees SB, Guentner A, Chey SW, Saad R, Chey WD. How Do US Gastroenterologists Use Over-the-Counter and Prescription Medications in Patients With Gastroesophageal Reflux and Chronic Constipation?. Am J Gastroenterol 2015, in press. 10.1038/ajg.2015.156 26054623

[12] Harari D, Halter JB, Ouslander JG, Tinetti ME (2009). Constipation. Hazzard’s Geriatric Medicine and Gerontology.

[13] Harris LA (2005). Prevalence and ramifications of chronic constipation. Manag Care Interface.

[14] Whitehead WE, Drinkwater D, Cheskin LJ, Heller BR, Schuster MMJ (1989). Constipation in the elderly living at home. Definition, prevalence, and relationship to lifestyle and health status. Am Geriatr Soc.

[15] Morley JE, Kim MJ, Haren MT, Kevorkian R, Banks WA (2005). Frailty and the aging male. Aging male.

[16] Wolfsen CR, Barker JC, Mitteness LS (1993). Constipation in the daily lives of frail elderly people. Arch Fam Med.

[17] Fox JC, Fletcher JG, Zinsmeister AR, Seider B, Riederer SJ (2006). Bharucha AE. Effect of aging on anorectal and pelvic floor functions in females. Dis Colon Rectum..

[18] Werta BL, Williams KA, Pont LG. A longitudinal study of constipation and laxative use in a community-dwelling elderly population. Arch Gerontol Gertiatr. 2015, in press 10.1016/j.archger.2015.02.004 25736738

[19] Crane SJ, Talley NJ (2007). Chronic gastrointestinal symptoms in the elderly. Clin Geriatr Med.

[20] Kimberly BS (2007). Constipation in the elderly: implication in skilled nursing facilities. Director.

[21] Coyne KS, Cash B, Kopp Z, Murphy S, Smith GJ, Davidson GA (2011). The prevalence of chronic constipation and fecal incontinence among men and women with symptoms of overactive bladder. BJU Int..

[22] Bouras EP, Tangalos EG (2009). Chronic constipation in the elderly. Gastroenterol Clin North Am.

[23] Kinnunen O (1991). Study of constipation in a geriatric hospital, day hospital, old people’s home and at home. Aging.

[24] Lim SF, Ong SY, Tan YL, Ng YS, Chan YH, Childs C. Incidence and predictors of new-onset constipation during acute hospitalisation after stroke. Int J Clin Pract 2015, in press doi:10.1111/ijcp.12528 10.1111/ijcp.12528 25656963

[25] Rao SS, Seaton K, Miller MJ (2007). Psychological profiles and quality of life differ between patients with dyssynergia and those with slow transit constipation. J Psychosom Res.

[26] O’Keefe EA, Talley NJ, Tangalos EG, Zinsmeister AR (1992). A bowel symptom questionnaire for the elderly. J Gerontol..

[27] Talley NJ (2004). Definitions, epidemiology, and impact of chronic constipation. Rev Gastroenterol Disord.

[28] Pare P, Ferrazzi S, Thompson WG, Irvine EJ, Rance L (2001). An epidemiological survey of constipation in Canada: definitions, rates, demographics, and predictors of health care seeking. Am J Gastroenterol..

[29] Bassotti G, Chiarioni G, Vantini I (1994). Anorectal manometric abnormalities and colonic propulsive impairment in patients with severe chronic idiopathic constipation. Dig Dis Sci.

[30] Cook IJ, Talley NJ, Benninga MA, Rao SS, Scott SM (2009). Chronic constipation: overview and challenges. Neurogastroenterol Motil..

[31] Rao SS (2007). Constipation: evaluation and treatment of colonic and anorectal motility disorders. Gastroenterol Clin North Am.

[32] Koch TR, Carney JA, Go VL, Szurszewski JH (1991). Inhibitory neuropeptides and intrinsic inhibitory innervation of descending human colon. Dig Dis Sci..

[33] Bassotti G, Villanacci V (2011). Can “functional” constipation be considered as a form of enteric neuro-gliopathy?. Glia.

[34] Camilleri M, Lee JS, Viramontes B, Bharucha AE, Tangalos EG (2000). Insights into the pathophysiology and mechanisms of constipation, irritable bowel syndrome, and diverticulosis in older people. J Am Geriatr Soc..

[35] Varma JS, Bradnock J, Smith RG, Smith AN (1988). Constipation in the elderly. A physiologic study. Dis Colon Rectum.

[36] Knowles CH, Farrugia G (2011). Gastrointestinal neuromuscular pathology in chronic constipation. Best Pract Res Clin Gastroenterol.

[37] Szurszewski JH, Holt PR, Schuster M (1989). Proceedings of a workshop entitled “Neuromuscular function and dysfunction of the gastrointestinal tract in aging”. Dig Dis Sci.

[38] Klosterhalfen B, Offner F, Topf N, Vogel P, Mittermayer C (1990). Sclerosis of the internal anal sphincter, a process of aging. Dis Colon Rectum..

[39] Papachrysostomou M, Pye SD, Wild SR, Smith AN (1994). Significance of the thickness of the anal sphincters with age and its relevance in fecal incontinence. Scand J Gastroenterol..

[40] Lovell R, Ford A (2012). Global prevalence of, and risk factors for, irritable bowel syndrome: a meta- analysis. Clin Gastroenterol Hepatol.

[41] Longstreth GF, Thompson WG, Chey WD, Houghton LA, Mearin F, Spiller RC (2006). Functional bowel disorders. Gastroenterology..

[42] Bassotti G, Maggio D, Battaglia E (2000). Manometric investigation of anorectal function in early and late stage Parkinson’s disease. J Neurol Neurosurg Psychiatry.

[43] Iovino P, Chiarioni G, Bilancio G (2013). New onset of constipation during long-term physical inactivity: a proof-of-concept study on the immobility-induced bowel changes. PLoS One.

[44] Spiller R, Camilleri M, Longstreth GF (2010). Do the symptom-based, Rome criteria of irritable bowel syndrome lead to better diagnosis and treatment outcomes?. Clin Gastroenterol Hepatol.

[45] Saad RJ, Rao SS, Koch KL (2010). Do stool form and frequency correlate with whole-gut and colonic transit? Results from a multicenter study in constipated individuals and healthy controls. Am J Gastroenterol.

[46] Chiarioni G, Kim SM, Vantini I, Whitehead WE (2014). Validation of the balloon evacuation test: reproducibility and agreement with findings from anorectal manometry and electromyography. Clin Gastroenterol Hepatol.

[47] Tack J, Müller-Lissner S, Stanghellini V (2011). Diagnosis and treatment of chronic constipation--a European perspective. Neurogastroenterol Motil..

[48] Bassotti G, Stanghellini V, Chiarioni G (1996). Upper gastrointestinal motor activity in patients with slow-transit constipation. Further evidence for an enteric neuropathy. Dig Dis Sci.

[49] Redmond JM, Smith GW, Barofsky I, Ratych RE, Goldsborough DC, Schuster MM (1995). Physiological tests to predict long-term outcome of total abdominal colectomy for intractable constipation. Am J Gastroenterol..

[50] Arora Z, Parungao JM, Lopez R, Heinlein C, Santisi J, Birgisson S (2015). Clinical Utility of Wireless Motility Capsule in Patients with Suspected Multiregional Gastrointestinal Dysmotility. Dig Dis Sci.

[51] Chiarioni G, Heymen S, Whitehead WE (2006). Biofeedback therapy for dyssynergic defecation. World J Gastroenterol.

[52] Andersson H, Bosaeus I, Falkheden T, Melkersson M (1979). Transit time in constipated geriatric patients during treatment with a bulk laxative and bran: a comparison. Scand J Gastroenterol..

[53] Nour-Eldein H, Salama HM, Abdulmajeed AA, Heissam KS (2014). The effect of lifestyle modification on severity of constipation and quality of life of elders in nursing homes at Ismailia city, Egypt. J Family Community Med.

[54] Ford AC, Moayyedi P, Lacy BE (2014). American College of Gastroenterology Monograph on the Management of Irritable Bowel Syndrome and Chronic Idiopathic Constipation. Am J Gastroenterol.

[55] Spinzi G, Amato A, Imperiali G (2009). Constipation in the elderly: management strategies. Drugs Aging.

[56] Suares NC, Ford AC (2011). Systematic review: the effects of fibre in the management of chronic idiopathic constipation. Aliment Pharmacol Ther.

[57] Fleming V, Wade WE (2010). A review of laxative therapies for treatment of chronic constipation in older adults. Am J Geriatr Pharmacother.

[58] Attaluri A, Donahoe R, Valestin J, Brown K, Rao SSC (2011). Randomised clinical trial: dried plums (prunes) vs. psyllium for constipation. Aliment Pharmacol Ther.

[59] Miller LE, Ouwehand AC (2013). Probiotic supplementation decreases intestinal transit time: meta-analysis of randomized controlled trials. World J Gastroenterol.

[60] Chiarioni G, Whitehead WE, Parkman HP, McCallum RW, Rao SSC (2011). Biofeedback therapy for constipation. GI Motility Testing: a Laboratory and Office Handbook.

[61] Simón MA, Bueno AM (2009). Behavioural treatment of the dyssynergic defecation in chronically constipated elderly patients: a randomized controlled trial. Appl Psychophysiol Biofeedback.

[62] Rao SSC, Go JT (2010). Update on the management of constipation in the elderly: new treatment options. Clin Interv Aging.

[63] Passmore AP, Davies KW, Flanagan PG, Stoker C, Scott MG (1993). A comparison of Agiolax and lactulose in elderly patients with chronic constipation. Pharmacology.

[64] Connolly P, Hughes IW, Ryan G (1975). Comparison of “Duphalac” and “irritant” laxatives during and after treatment of chronic constipation: a preliminary study. Curr Med Res Opin.

[65] Lederle FA, Busch DL, Mattox KM, West MJ, Ask DM (1990). Cost-effective treatment of constipation in the elderly. A randomized double-blind comparison of sorbitol and lactulose. Am J Med.

[66] Miller LE, Tennilä J, Ouwehand AC (2014). Efficacy and tolerance of lactitol supplementation for adult constipation: a systematic review and meta-analysis. Clin Exp Gastroenterol.

[67] Ouwehand AC, Tiihonen K, Saarinen M, Putaala H, Rautonen N (2009). Influence of a combination of Lactobacillus acidophilus NCFM and lactitol on healthy elderly: intestinal and immune parameters. Br J Nutr.

[68] De Giorgio R, Cestari R, Corinaldesi R (2011). Use of macrogol 4000 in chronic constipation. Eur Rev Med Pharmacol Sci.

[69] DiPalma JA, Cleveland MV, McGowan J, Herrera HL (2007). A randomized, multicenter, placebo-controlled trial of polyethylene glycol laxative for chronic treatment of chronic constipation. Am J Gastroenterol.

[70] Corazziari E, Badiali D, Bazzocchi G (2000). Long term efficacy, safety, and tolerabilitity of low daily doses of isosmotic polyethylene glycol electrolyte balanced solution (PMF-100) in the treatment of functional chronic constipation. Gut.

[71] DiPalma JA, Cleveland MB, McGowan J, Herrera HL (2007). A comparison of polyethylene glycol laxative and placebo for relief of constipation from constipating medications. South Med J.

[72] Chapman RW, Stanghellini V, Geraint M, Halphen M (2013). Randomized clinical trial: macrogol/PEG 3350 plus electrolytes for treatment of patients with constipation associated with irritable bowel syndrome. Am J Gastroenterol.

[73] Belsey JD, Geraint M, Dixon TA (2010). Systematic review and meta analysis: polyethylene glycol in adults with non-organic constipation. Int J Clin Pract.

[74] Crowell MD, Harris LA, Lunsford TN, Dibaise JK (2009). Emerging drugs for chronic constipation. Expert Opin Emerg Drugs..

[75] Crowell MD, Harris LA, Dibaise JK, Olden KW (2007). Activation of type-2 chloride channels: a novel therapeutic target for the treatment of chronic constipation. Curr Opin Investig Drugs..

[76] Lacy BE, Levy LC (2008). Lubiprostone: a novel treatment for chronic constipation. Clin Interv Aging.

[77] Barish CF, Drossman D, Johanson JF, Ueno R (2010). Efficacy and safety of lubiprostone in patients with chronic constipation. Dig Dis Sci..

[78] Johanson JF, Morton D, Geenen J, Ueno R (2008). Multicenter, 4-week, double-blind, randomized, placebo-controlled trial of lubiprostone, a locally-acting type-2 chloride channel activator, in patients with chronic constipation. Am J Gastroenterol..

[79] Johanson JF, Ueno R (2007). Lubiprostone, a locally acting chloride channel activator, in adult patients with chronic constipation: a double-blind, placebo-controlled, dose-ranging study to evaluate efficacy and safety. Aliment Pharmacol Ther.

[80] Gras-Miralles B, Cremonini F (2013). A critical appraisal of lubiprostone in the treatment of chronic constipation in the elderly. Clin Interv Aging.

[81] Layer P, Stanghellini V (2014). Review article: Linaclotide for the management of irritable bowel syndrome with constipation. Aliment Pharmacol Ther.

[82] Lacy BE, Levenick JM, Crowell M (2012). Chronic constipation: new diagnostic and treatment approaches. Ther Adv Gastroenterol.

[83] Lembo AJ, Kurtz CB, Macdougall JE (2010). Efficacy of linaclotide for patients with chronic constipation. Gastroenterology.

[84] Rao SS, Quigley EM, Shiff SJ (2014). Effect of Linaclotide on Severe Abdominal Symptoms in Patients with Irritable Bowel Syndrome with Constipation. Clin Gastroenterol Hepatol.

[85] De Giorgio R, Barbara G, Furness JB, Tonini M (2007). Novel therapeutic targets for enteric nervous system disorders. Trends Pharmacol Sci..

[86] Quigley EMM (2012). Prucalopride: safety, efficacy and potential applications. Ther Adv Gastroenterol.

[87] Tack J, Stanghellini V, Dubois D, Joseph A, Vandeplassche L, Kerstens R (2014). Effect of prucalopride on symptoms of chronic constipation. Neurogastroenterol Motil.

[88] Muller-Lissner S, Rykx A, Kerstens R, Vandeplassche L (2010). A double-blind, placebo-controlled study of prucalopride in elderly patients with chronic constipation. Neurogastroenterol Motil.

[89] Camilleri M, Beyens G, Kerstens R, Robinson P, Vandeplassche L (2009). Safety assessment of prucalopride in elderly patients with constipation: A double blind, placebo-controlled study. Neurogastroenterol Motil..

[90] Goldberg M, Li YP, Johanson JF (2010). Clinical trial: the efficacy and tolerability of velusetrag, a selective 5-HT4 agonist with high intrinsic activity, in chronic idiopathic constipation -a 4-week, randomized, double-blind, placebo-controlled, dose–response study. Aliment Pharmacol Ther.

[91] Camilleri M, Vazquez-Roque MI, Burton D (2007). Pharmacodynamic effects of a novel prokinetic 5-HT receptor agonist, ATI-7505, in humans. Neurogastroenterol Motil.

[92] Chey WD, Camilleri M, Chang L, Rikner L, Graffner H (2011). A randomized placebo-controlled phase IIb trial of A3309, a bile acid transporter inhibitor, for chronic idiopathic constipation. Am J Gastroenterol..

[93] Shailubhai K, Comiskey S, Foss JA (2013). Plecanatide, an oral guanylate cyclase C agonist acting locally in the gastro- intestinal tract, is safe and well-tolerated in single doses. Dig Dis Sci.

[94] Miner PB, Surowitz R, Fogel R (2013). Plecanatide, a novel guanylate-cyclase C (GC-C) receptor agonist, is efficacious and safe in patients with chronic idiopathic constipation (CIC): results from a 951 patient, 12 week, multi-center trial. Digestive Diseases Week.

